# Characterization of pork patties containing dry radish (*Raphanus sativus*) leaf and roots

**DOI:** 10.5713/ajas.18.0384

**Published:** 2018-08-27

**Authors:** Su-Jin Ahn, Hyung Joo Kim, Nayeon Lee, Chi-Ho Lee

**Affiliations:** 1 Department of Food Science and Biotechnology of Animal Resources, Konkuk University, Seoul 143-701, Korea; 2 National Forensic Service, Wonju 26460, Korea

**Keywords:** Radish Leaves, Pork, Patties, Quality, Refrigeration

## Abstract

**Objective:**

This study investigated the effects of dry radish leaf and root on the quality of pork patties during refrigeration storage.

**Methods:**

The patties were divided into the following three groups: the control containing 0% dry radish leaf root powder, RL1 containing 0.5% dry radish leaf root powder, and RL2 and RL3 containing 1% and 2% dry radish leaf root powder, respectively. Proximate composition, pH, cooking loss, microbial analysis, lipid oxidation analysis, color, texture profile analysis and sensory test were performed.

**Results:**

Moisture, crude protein, and crude ash contents in RL2 and RL3 were significantly higher than those in other groups (p<0.05), whereas crude fat contents in RL2 and RL3 were significantly lower than other groups (p<0.05). Lightness was significantly lower in RL2 and RL3 than in CON (p<0.05). Cooking loss for RL2 and RL3 were significantly lower than those for the other groups (p<0.05). The pH, thiobarbituric acid levels, and total plate counts of RL2 and RL3 were significantly lower than those of CON at days 7 and 14 (p<0.05). Hardness values of RL2 and RL3 were significantly lower than those of CON, whereas chewiness values were higher than those of CON (p<0.05). In addition, the juiciness of RL2 were significantly greater (p<0.05) than those of the other groups.

**Conclusion:**

Dried radish leaves and roots improved the proximate composition and quality characteristics of pork patties, providing a basis to produce high-quality patties with extended expiration dates. Thus, dried radish leaves and roots are effective ingredients for health or functional foods.

## INTRODUCTION

In South Korea, the westernization of food culture and fast food consumption are rapidly increasing. Similarly, the consumption of patties is continuously increasing but intemperate intake of meat can cause not only obesity but also a variety of adult diseases. Meat and meat products including beef and pork are rich in high-quality protein providing essential amino acids and various vitamins; they have excellent nutritional value, high flavor, and Hamburger patties are a typical fast food; they are made from minced meat, such as beef or pork, and other ingredients, such as bread crumbs and eggs. Although meat and meat products, including beef and pork, are rich in high-quality protein and have excellent nutritional value, flavor, and aroma, the fats, salt, and additives in burger patties are unhealthy. Accordingly, consumers have demanded healthy ingredients in patties [1]. Recently, the development of patties with various healthy and functional ingredients has increased, with reductions in unhealthy compounds, e.g., fats, salt, and other additives.

Functional food refers to foods prepared by the addition of natural materials with functional ingredients to produce a positive function *in vivo* after ingestion. Consumers are increasingly interested in functional foods. These functional foods must have satisfactory quality characteristics, such as taste and nutrition, while providing useful functions. Dietary fiber from various natural materials is a functional ingredient with beneficial physiological effects; it has been included in meat products, including patties [2].

Radish (*Raphanus sativus* L.) leaves and roots contain a large amount of fiber as well as various vitamins and minerals; thus, they are utilized as a source of dietary fiber [3]. During the past few decades, several studies have examined the effects of the consumption of cruciferous vegetables on health, particularly the polyphenolic profile and antioxidant properties of radish leaves and roots. Radish leaves have high vitamin A, vitamin C, and fiber contents and accordingly are promising for functional food products [4]. In addition to the β-carotene content, the potassium and calcium contents in soluble dietary fiber are also high. Radish leaves also exert an antihypertensive effect in spontaneously hypertensive rats, which results from reduced angiotensin-converting enzyme (ACE) activity and increased urinary Na excretion [5]. Moreover, dietary fibers have additional physiological effects, such as low density lipoprotein cholesterol reduction, cancer prevention, and mutation suppression [6].

Previous reports have shown enhanced quality characteristics and delays in retrogradation when radish leaves are added to Seolgiddeok [7]. Recently, various modifications to patties have been reported, such as the development of yam powder-containing meat products [8]. In addition, dietary fiber-rich and low-fat ingredients, such as low-calorie seaweed, have been added to pork patties [9].

These previous findings suggest that the development and characterization of processed meat products containing radish leaves as an additive are important research goals owing to the growing interest in functional foods. In this study, we investigated the effects of these additives on the quality and sensory characteristics of pork patties to obtain basic data for the use of pork meat in the manufacture of meat products.

## MATERIALS AND METHODS

### Preparation of products

Dry radish leaves from the demilitarized zone (DMZ) punchbowl region (Yanggu, Gangwon Province, Korea) were used for patty production and other experiments. The formulation of pork patties containing dry radish leaf and root is shown in Table 1. Lean pork meat and pork back fat were bought from a local market (Seoul, Korea). Fat and connective tissue were removed from the pork meat. Lean pork meat and fat were ground through a 5-mm plate. Dry radish leaves and root were ground in a blender and added in powder form. Pork meat was homogenized and ground for 1 min in a silent cutter (Cutter N.-963009, Scharfen, Germany). Garlic powder (0.05%) and sodium nitrite (0.015%; Seoul, Korea), previously dissolved in water, were chilled (2°C), and then added to the pork meat mixture and mixed for 1 min. After mixing, the dry radish leaves and root were added at 0.5%, 1%, or 2%. Mixtures with different compositions were each mixed and formed into patties (100±1 g each), using 15×90 mm sterilized Petri dishes. The patties were then heated at 75°C for 45 min in a water bath (model 10-101, Dae Han, Co., Seoul, Korea). Patties were used for the experiments after cooking on a daily basis for 0, 3, 7, and 14 d. The samples were then stored at 4°C for 0, 3, 7, and 14 d in a refrigerator.

### Proximate composition of dry radish leaf and root patties

Compositional properties of the cured pork lean meat were determined according to the criteria of the Association of Official Analytical Chemists (AOAC) [10]. Crude protein and fat contents were determined by the Kjeldahl and Soxhlet methods, respectively. Moisture content was determined at 105°C under normal pressure by the drying method, whereas crude ash content was measured at 550°C by the direct ash method.

### pH determination

To determine the pH of the samples, 2 g of each sample was homogenized for 60 s in 18 mL of distilled water, using a bag mixer 400 (Interscience Co., Saint-Nom-la-Bretèche, France). The pH of the suspension was then measured using a pH meter (pH 900, Precisa, Dietikon, Switzerland).

### Determination of weight loss after cooking

The cooking loss (%) was determined using the weight differences in pork patties meat before and after cooking (steam cooking method with core temperature at 75°C for 45 min in a water bath), as shown below [11]:

Cooking loss (%)=(initial sample weight-sample weight after cooking)/initial sample weight×100

### Microbial analysis

For microbial analysis, 2 g of each sample was homogenized in 18 mL of sterile 0.85% NaCl solution for 90 s with a bag mixer and inoculated onto Petrifilm (3M, St. Paul, MN, USA). Total counts of aerobic bacteria were obtained after 24 h of incubation at 36°C.

### Lipid oxidation analysis

Lipid oxidation was determined by the 2-thiobarbituric acid (TBARS) method described by Kim et al [12]. First, 2 g of sample was homogenized at 12,000 rpm for 1 min in 10 mL of 10% trichloroacetic acid and 10 mL of distilled water. After homogenization, the solution was filtered through filter paper (Whatman No. 1, Whatman, Piscataway, NJ, USA). Subsequently, 5 mL of filtered solution was mixed with 5 mL of 2.88 g/L TBARS, and the mixed solution was placed in a 90°C water bath for 10 min. After 30 min of cooling, the absorbance of the solution at 532 nm was determined using a spectrophotometer (UV/Vis Spectrophotometer, Mecasys, Daejeon, Korea). Data are shown as mg of malondialdehyde (MDA) meat/kg. Standard MDA curves determined from 1,1,3,3-tetraethoxypropane were used for calculations.

### Color determination

The color of each pork mixture preparation was determined using a colorimeter (Minolta Chroma meter CR-210, Japan; illuminate C, standard with a white plate, Commission Internationale de l’Eclairage (CIE) L* = +97.83, CIE a* = −0.43, CIE b* = +1.98) in triplicate. Lightness (CIE L*-value), redness (CIE a*-value), and yellowness (CIE b*-value) values were obtained.

### Texture profile analysis

Texture of the samples used in the cooking loss analysis was determined. After heat processing, the patties were placed in a texture analyzer (CT3-1000, Brookfield Engineering Laboratories, Middleboro, MA, USA). Texture was calculated using the values from two consecutive compressions at the sample center with a circular probe (TA418-Sphere, 12.7 mmd). The samples were prepared as 1.50 cm×1.50 cm×1.50 cm blocks with a trigger load of 10 g at a crosshead speed of 1.0 mm/s. Texture was measured 5 consecutive times to determine hardness, cohesiveness, springiness, gumminess, and chewiness.

### Sensory test

Sensory tests of patties with or without dry radish leaf and root were performed. Overall acceptability scores (1 = not acceptable and 10 = very acceptable) were given by 10 randomly assigned trained panelists (6 women and 4 men aged 23 to 29 years with an average age of 26.2 years) from Konkuk University for sensory evaluation. Patties were cut into blocks with a thickness of 1 cm, length of 1.5 cm, and width of 1.5 cm and were provided on a white plate at room temperature (18°C to 21°C). After eating from one sample, panelists were asked to rinse their mouths with water and eat the next sample after waiting 1 to 2 min for the evaluation. Scores for meat color, flavor, tenderness, juiciness, and overall acceptability were provided by the tasters. The sensory description was explained to the panelists by the method described by Li et al [13].

### Statistical analysis

The experiments were designed to have two types of samples (CON and RL). Averages and standard deviations of test results with 95% confidence intervals are presented. The SPSS program (Version 12.0, SPSS Inc., Chicago, IL, USA) was used for each test. Tukey’s multiple range test was performed to determine statistically significant differences (p<0.05).

## RESULTS AND DISCUSSION

### Effect of adding dry radish on the proximate composition of patties

The proximate compositions of patties with or without dried radish leaves and roots are shown in Table 2. The moisture and crude ash contents in RL2 and RL3 were higher than those in other groups (p<0.05). The dietary fiber in pork products increases the moisture content by improving the water-holding capacity, thereby reducing cooking loss [14]. Additionally, the increase in ash corresponded to the radish mineral composition. The crude protein contents in the treatment groups were significantly greater than that in the CON group (p<0.05). The crude protein content of the dried radish resulted in an increase in the crude protein content in pork patties containing dried radish leaves and roots. The crude fat content was lower in RL2 and RL3 than in the CON group (p<0.05). A similar reduction in the fat content has been reported by Kim et al [15]. Our results showed that radish leaves and roots increased the moisture, crude protein, and crude ash contents of pork patties and decreased the crude fat content; therefore, this approach can be used to produce healthy meat products.

### Effect of adding dry radish on pH during storage of patties

**Figure 1:**
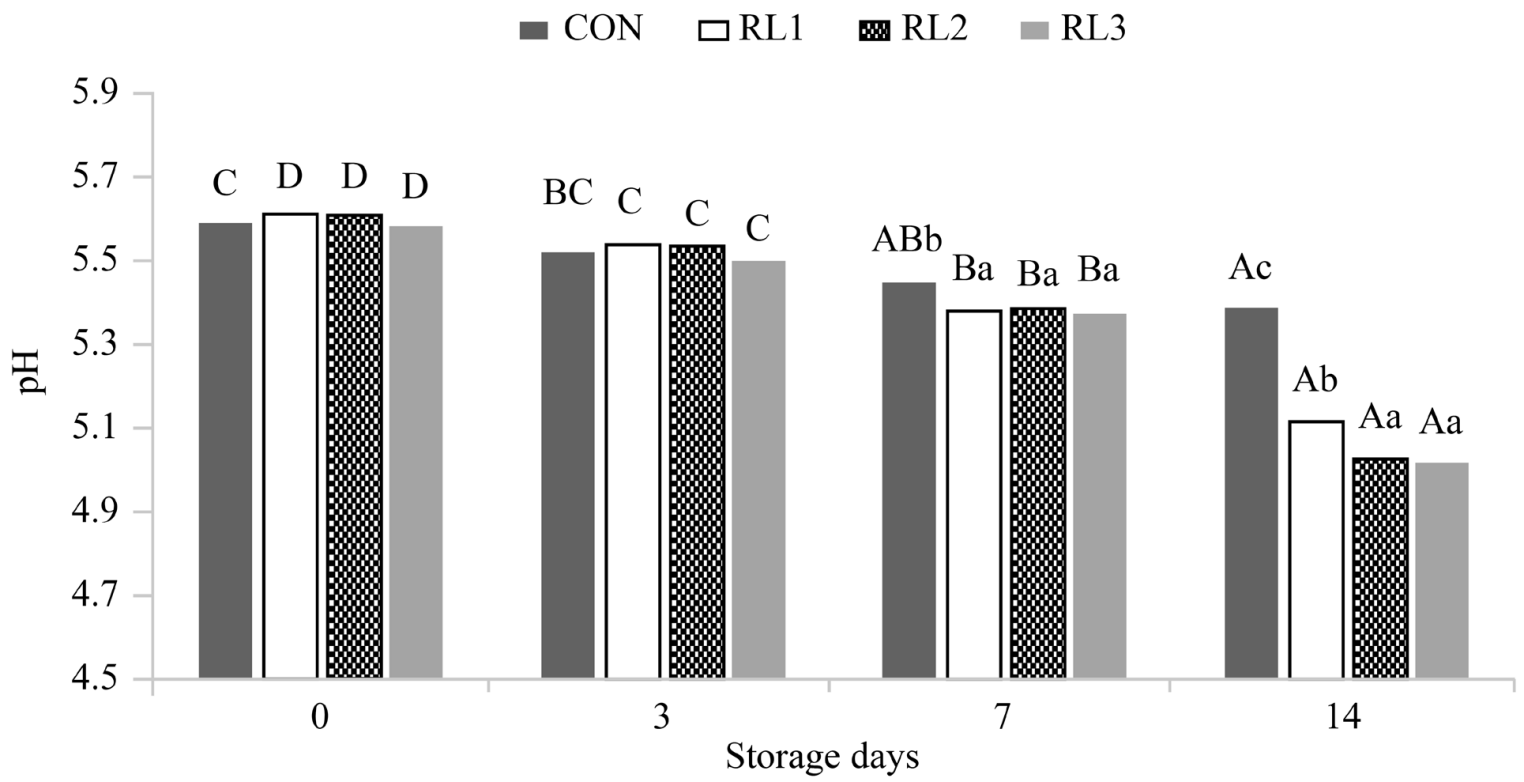
pH changes in pork patties with or without dry radish leaf and root during storage at 4°C for 14 days. Values correspond to the mean±standard deviation (n = 3). CON, control (no addition); RLI, addition of 0.5% radish leaves and root; RL2, addition of 1% radish leaves and root; RL3, addition of 2% radish leaves and root. ^A–D^ Values sharing different letters in the same treatment are significantly different (p<0.05). ^a–c^ Values sharing different letters in the same storage duration are significantly different (p<0.05).

The pH values of patties stored for 14 d are shown in Figure 1. No significant difference was observed since day 0 until day 3 in all samples. However, the pH values of all samples decreased significantly during storage (p<0.05). The pH values were significantly lower in the treatment groups than in CON at days 7 and 14 (p<0.05). The low pH value in pork products influences various factors during storage, such as the shelf life, loss in redness over time, quality characteristics, water-binding capacity, and texture. In addition, low pH values in pork products can inhibit the growth of microorganisms and reduce the content of biogenic amines [16]. Therefore, the lower pH values of RL2 and RL3 compared to that of the control group may be associated with reduced antimicrobial activity.

The pH values of all samples decreased during storage, but those of refrigerated products, such as meat products, typically decrease according to the refrigeration time or the accumulation of lactic acid. Vegetables (such as radishes) increase lactic acid fermentation by promoting the growth of lactic acid bacteria [17]. Therefore, the addition of dried radish leaves and roots reduces the pH, prevents microbial growth, and affects lipid oxidation.

### Effect of adding dry radish on the cooking loss of patties

The cooking loss of pork patties with or without dry radish leaves and roots is shown in Table 3. The cooking loss for all groups increased significantly at day 14 (p<0.05). The cooking losses were lower in RL2 and RL3 than in other groups at all day (p<0.05). Similar trends in cooking loss after the addition of dietary fiber to pork products have been reported previously [18]. This reduction in cooking loss results from the high water-binding capacity and high moisture absorption of the dietary fiber; cooking loss is also affected by the fat and water contents [14].

### Effect of adding dry radish on microbial activity in patties

**Figure 2:**
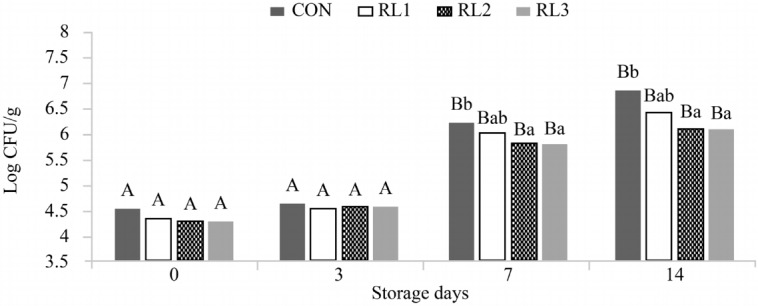
Microbiological changes in pork patties with or without dry radish leaf and root during storage at 4°C for 14 days. Values correspond to the mean±standard deviation (n = 6). CON, control (no addition); RLI, addition of 0.5% radish leaves and root; RL2, addition of 1% radish leaves and root; RL3, addition of 2% radish leaves and root. ^A,B^ Values sharing different letters in the same treatment are significantly different (p<0.05). ^a,b^ Values sharing different letters in the same storage duration are significantly different (p<0.05).

Microbiological changes during the storage period are shown in Figure 2. The total plate counts (TPC) for all groups increased significantly at days 7 and 14 (p<0.05). At days 7 and 14, RL2 and RL3 showed significantly lower TPCs than that for the CON group (p<0.05) and the lowest TPCs among all groups (p<0.05). Microorganisms damage lipids in a process called lipid oxidation. Sahoo and Anjaneyulu have demonstrated that there is a positive correlation between microbiological parameters and TBARS [19].

Various underlying mechanisms contribute to the antimicrobial activity of natural materials. Phenols and flavonoids bind to substances that are indispensable for the metabolism of microorganisms. The destruction of the cell membrane has an antibacterial effect. Our results are consistent with those of a previous study that reported antimicrobial activity in radish leaves before blanching [20].

These results suggest that the addition of radish leaves prevented microbial spoilage in pork patties, consistent with the findings of Lee et al [21]. Additionally, the low pH value effectively inhibited the growth of microorganisms.

### Effect of adding dry radish on lipid oxidation in patties

**Figure 3:**
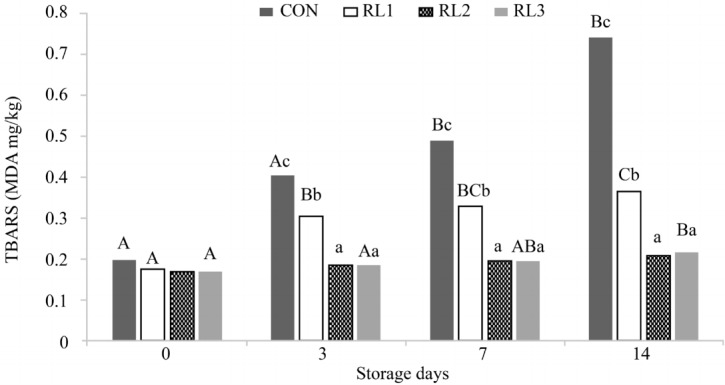
Changes in TBARS value of pork patties with or without dry radish leaf and root during storage at 4°C for 14 days. Values correspond to the mean±standard deviation (n = 5). CON, control (no addition); RLI, addition of 0.5% radish leaves and root; RL2, addition of 1% radish leaves and root; RL3, addition of 2% radish leaves and root. ^A–C^ Values sharing different letters in the same treatment are significantly different (p<0.05). ^a–c^ Values sharing different letters in the same storage duration are significantly different (p<0.05).

To evaluate the effect of dried radish on lipid oxidation in patties, the TBARS values of pork patties were determined during storage at 4°C±1°C for 14 d (Figure 3). The TBA values of RL1, RL3, and CON increased significantly during storage (p<0.05), consistent with the results of a previous study [22]. There were no significant differences in TBARS values among groups at day 0 (p<0.05). Thereafter, during the storage period, the TBA values of RL2 and RL3 were significantly lower than those of the CON group (p<0.05). The antioxidant activities of radish leaves have been attributed to esters and flavonoids, as the main phenolic compounds [23]. The TBA values of high-quality meat products are approximately 0.2 mg MDA/kg. Sensory quality degradation begins when TBARS values reach 0.46 mg MDA/kg, and an outright putrefaction state occurs at 1.2 mg MDA/kg. In this experiment, when estimating rancidity on the basis of the TBARS value of 0.4 mg MDA/kg, RL2 and RL3 maintained a high quality until day 14. Additionally, Naveena et al [24] reported that treatment with lactic acid prevents increases in TBARS and microbial counts without influencing color and odor. These findings are in accordance with studies indicating that radishes can be used as natural antioxidants to enhance the storage stability of pork-based meat products.

### Effect of adding dry radish on color changes in patties

Table 4 shows the color properties of the sample groups. The lightness (L*) was significantly lower in RL2 and RL3 than in the other groups (p<0.05). There were no significant differences in a* values among samples. However, the a* value decreased significantly in the treatment groups during storage (p<0.05) but not in the CON group. Phillips et al [25] determined that the decrease in redness during storage was closely related to lipid oxidation. Color stability can differ according to muscle type, since each muscle has a distinct oxygen consumption rate and metmyoglobin reductase activity. The b* values in RL3 were significantly higher than those of other groups at 0 day (p<0.05). Additionally, the b* values of RL1 and RL2 increased significantly during the storage period (p<0.05).

Previous reports have indicated that that L* increases as lipid oxidation increases during processing [12]. In general, the color stability of meat appears to be related to lipid oxidation. Therefore, the lower L* values in pork patties containing dried radish leaves and roots could be explained by a reduction in lipid oxidation.

The color changes were affected by the color of the additives themselves. These results were similar to those of other studies, particularly studies of meat products with citrus peel powder [26]. Dried radish contains chlorophyll, and it is thought that the green color is reflected in the meat products containing it as an additive.

### Effect of adding dry radish on the textural properties of patties

Texture profile analysis (TPA) is the most frequently utilized technique for analyzing the textural properties of foods. Hardness, cohesiveness, gumminess, and chewiness are related to the water activity and moisture contents of food. Table 5 shows the results of TPA determination in pork patties with or without dried radish leaves and roots. The hardness of treatment groups was significantly lower than that of CON (p<0.05). Previous studies have suggested that the meat protein system can explain the softer texture after the addition of non-meat ingredients [27]. Additionally, these results may be associated with the water binding properties of radish leaves. Our results were consistent with those of Yang et al [28]. However, the chewiness of RL2 and RL3 was significantly higher than those of the other groups (p<0.05). These results were consistent with those of Jeon and Choi [9], who reported that the chewiness of patties increases by the addition of seaweed and that this property can be attributed to the binding ability of dietary fiber.

However, there were no significant differences in cohesiveness, springiness, and gumminess among groups, in contrast to results of Yang et al [28].

### Effect of adding dry radish on the sensory evaluation of patties

The results of a sensory evaluation, a quality index related to physiochemical characteristics, are shown in Table 6. The juiciness of RL2 was significantly higher than those of the other groups (p<0.05). These results are consistent with those of Desmond et al [29], who found that dietary fiber improves water holding in manufactured meat patties. Dietary fiber also retains moisture and, when cooking is complete, prevents the meat from drying. Juiciness is related to the moisture content, which is an important determinant of meat quality.

In this study, the effects of dried radish leaves and roots on the quality of pork patties during refrigerated storage were investigated. Dried radish leaves and roots were added at 0%, 0.5%, 1%, and 2% to pork patties and quality characteristics were evaluated. TPCs for patties containing dried radish leaves and roots were lower than those of the control during storage, especially for RL2 and RL3. Additionally, the overall acceptability of RL2 was significantly higher than that of the other groups. The addition of 1% dried radish leaves and roots to pork patties can improve the microbial storage stability and overall preference.

In conclusion, adding dried radish leaves and roots to pork patties is expected to improve sensory properties. In particular, the addition of 1% and 2% dried radish leaves and roots to pork patties improves the moisture content, crude fat content, microbiological properties, and TBARS value. The addition of 1% dried radish leaves and roots to pork patties is beneficial with respect to cost and quality according to the results of this study.

## Figures and Tables

**Table 1:** Formulation (%) of pork patties containing dry radish leaf and root

Ingredients	Samples1)			

	CON	RL1	RL2	RL3
Lean pork meat	70	70	70	70
Pork back fat	10	10	10	10
Water	20	20	20	20
Total	100	100	100	100
Salt	2	2	2	2
Black pepper	0.05	0.05	0.05	0.05
Garlic powder	0.05	0.05	0.05	0.05
Sodium nitrite	0.015	0.015	0.015	0.015
Glucose	1	1	1	1
Radish leaf and root	0	0.5	1	2

1) CON, control (no addition); RL1, addition of 0.5% radish leaves and root; RL2, addition of 1% radish leaves and root; RL3, addition of 2% radish leaves and root.

**Table 2:** Proximate composition (%) of pork patties with or without dry radish leaf and root

Item	CON1)	RL11)	RL21)	RL31)
Crude ash (%)	1.83±0.01^a^	1.89±0.02^a^	2.02±0.01^b^	2.05±0.04^b^
Moisture (%)	71.25±0.13^a^	72.15±0.70^a^	74.18±0.72^b^	74.34±0.34^b^
Crude protein (%)	11.45±0.08^a^	13.89±0.02^b^	14.76±0.62^c^	14.99±0.06^c^
Crude fat (%)	11.74±0.03^c^	11.42±0.02^b^	10.91±0.06^a^	10.87±0.08^a^

Values are shown as the mean±standard deviation (n = 3).

1) CON, control (no addition); RL1, addition of 0.5% radish leaves and root; RL2, addition of 1% radish leaves and root; RL3, addition of 2% radish leaves and root.

Dry radish leaf and root composition (%): ash, 17.67; moisture, 36.46; protein, 4.28; gat, 1.86; dietary fiber, 39.73.

Mineral/vitamin, mg/g, dry base: Ca, 10.69 mg; P, 2.61 mg; Fe, 1.72 mg; Cu, 0.27 mg; Zn, 1.24 mg; Mn, 1.72 mg; K, 43.36 mg; Na, 3.62 mg; vitamin B1, 0.03 mg; vitamin B2, 0.06 mg; and vitamin C, 1,683.32 IU/kg.

a–c Values with different letters in the same row are significantly different (p<0.05).

**Table 3:** Changes in cooking loss (%) of pork patties with or without dry radish leaf and root during storage at 4°C for 14 d

Storage period (d)	Samples1)			

	CON	RL1	RL2	RL3
0	23.87±0.39^A^^b^	22.81±0.84^A^^ab^	21.74±0.63^A^^a^	21.14±0.83^A^^a^
3	24.95±0.54^A^^c^	23.27±0.14^A^^b^	21.91±0.41^A^^a^	21.90±0.30^A^^a^
7	25.07±0.80^A^^b^	24.63±0.65^AB^^b^	22.02±0.34^A^^a^	21.95±0.50^A^^a^
14	28.31±1.15^B^^b^	26.23±1.00^B^^ab^	25.90±0.29^B^^a^	25.65±0.41^B^^a^

Values are shown as the mean±standard deviation (n = 3).

1) CON, control (no addition); RL1, addition of 0.5% radish leaves and root; RL2, addition of 1% radish leaves and root; RL3, addition of 2% radish leaves and root.

A–B Values with different letters in the same row are significantly different (p<0.05).

a–c Values with different letters in the same column are significantly different (p<0.05).

**Table 4:** Color changes in pork patties with or without dry radish leaf and root during storage at 4°C for 14 d

Parameter	Storage period (d)	Samples1)			

		CON	RL1	RL2	RL3
L* (lightness)	0	55.33±1.06^b^	54.97±0.89^B^^b^	48.82±0.88^A^^a^	48.41±0.45^A^^a^
	3	56.22±0.55^c^	55.13±0.49^B^^b^	49.40±0.49^A^^a^	49.05±0.71^AB^^a^
	7	55.14±0.91^c^	53.38±0.85^A^^b^	49.46±0.83^A^^a^	50.03±0.77^B^^a^
	14	55.65±0.47^c^	54.07±0.70^AB^^b^	51.62±0.52^B^^a^	51.40±0.66^C^^a^
a* (redness)	0	4.87±0.09	4.97±0.10^B^	4.84±0.04^B^	4.85±0.13^B^
	3	4.84±0.13	4.81±0.24^B^	4.85±0.09^B^	4.86±0.12^B^
	7	4.86±0.16	4.73±0.25^AB^	4.75±0.11^B^	4.81±0.15^B^
	14	4.59±0.30	4.40±0.15^A^	4.42±0.15^A^	4.44±0.05^A^
b* (yellowness)	0	10.78±0.30^A^^a^	10.80±0.41^A^^a^	11.00±0.14^A^^a^	11.88±0.58^b^
	3	11.07±0.24^AB^^a^	11.12±0.40^AB^^ab^	11.28±0.08^AB^^ab^	11.57±0.26^b^
	7	11.71±0.32^C^	11.60±0.23^BC^	11.67±0.22^BC^	12.16±0.75
	14	11.63±0.32^BC^	11.76±0.47^C^	11.97±0.29^C^	12.01±0.63

Values are shown as the mean±standard deviation (n = 5).

1) CON, control (no addition); RL1, addition of 0.5% radish leaves and root; RL2, addition of 1% radish leaves and root; RL3, addition of 2% radish leaves and root.

a–c Values with different letters in the same row are significantly different (p<0.05).

A–C Values with different letters in the same column are significantly different (p<0.05).

**Table 5:** Texture profiles of pork patties with or without dry radish leaf and root during storage at 4°C for 14 d

Mechanical properties	Samples1)			

	CON	RL1	RL2	RL3
Hardness (g)	1,825.00±102.83^b^	1,664.20±88.60^a^	1,604.20±54.17^a^	1,596.00±95.62^a^
Cohesiveness	0.46±0.00	0.45±0.00	0.45±0.00	0.45±0.00
Springiness (mm)	6.64±0.05	6.66±0.04	6.62±0.01	6.60±0.05
Gumminess (g)	510.14±3.20	524.60±8.63	523.50±6.40	521.94±11.64
Chewiness (mJ)	33.80±4.68^a^	37.64±1.37^ab^	40.86±2.02^b^	41.32±2.41^b^

Values are shown as the mean±standard deviation (n = 5).

1) CON, control (no addition); RL1, addition of 0.5% radish leaves and root; RL2, addition of 1% radish leaves and root.

a–c Values with different letters in the same row are significantly different (p<0.05).

**Table 6:** Changes in sensory characteristics of cooked pork patties with or without dry radish leaf and root during storage at 4°C for 7 d

Parameter	Samples1)			

	CON	RL1	RL2	RL3
Color	3.30±1.16	3.30±1.06	3.20±1.14	3.10±0.88
Flavor	2.90±1.51	3.18±1.50	3.36±0.97	3.27±1.01
Tenderness	3.20±1.48	3.30±1.57	4.08±.1.93	3.88±1.36
Juiciness	3.27±1.01^a^	4.09±1.38^ab^	4.81±1.17^c^	4.45±1.37^ab^
Overall acceptability	3.90±1.30^a^	4.36±1.69^bc^	5.45±0.82^c^	4.64±1.12^bc^

Values are shown as the mean±standard deviation (n = 10).

1) CON, control (no addition); RL1, addition of 0.5% radish leaves and root; RL2, addition of 1% radish leaves and root; RL3, addition of 2% radish leaves and root.

a–c Values with different letters in the same row are significantly different (p<0.05).
